# Impact of Sjögren’s disease and its immunological characteristics on reaching remission or low disease activity state in systemic lupus erythematosus patients: a propensity score-matched longitudinal study

**DOI:** 10.3389/fimmu.2025.1639252

**Published:** 2025-09-29

**Authors:** Haoze Zhang, Huijuan Zhang, Dai Gao, Lanlan Ji, Yanjie Hao, Zhuoli Zhang

**Affiliations:** Department of Rheumatology and Clinical Immunology, Peking University First Hospital, Beijing, China

**Keywords:** systemic lupus erythematosus, remission on treatment, lupus low disease activity state, overlapping syndrome, Sjogren’ s disease

## Abstract

**Background:**

Systemic lupus erythematosus (SLE) overlapping with Sjögren’s disease (SjD) or not may progress differently in the clinical course. We aimed to explore the impact of SjD on lupus low disease activity state (LLDAS) or remission achievement in a real-world cohort.

**Methods:**

The medical records of patients in the Peking University First Hospital SLE (PKUFHS) cohort from 2007 to 2019 were retrospectively reviewed. Demographics, SLE and SjD features, treatment, and whether in LLDAS/remission on treatment (RONT) or not at each visit were collected. According to overlapped SjD and its immunological features, all patients were categorized into the following subgroups: SjD with anti-SSA single positivity, SjD with anti-SSA/SSB double positivity, SjD with baseline hypergammaglobulinemia, and those without SjD. The Cox proportional hazards model in propensity score-matched cohorts was used to estimate the impact of different SjD characteristics on LLDAS/RONT and each component after correcting for known confounders.

**Results:**

A total of 9,415 visits originating from 626 SLE patients were included. Overlapping SjD was identified in 77 (12.3%) patients. Patients with SLE overlapping SjD were significantly older at onset and predominantly female with more frequent hematological involvement. Overlapping SjD and anti-SSA/SSB double-positive SjD were associated with 29%–38% and 50%–53% reduction, respectively, in RONT achievement in SLE patients. Both overlapping SjD and SjD with baseline hypergammaglobulinemia acted as protectors for LLDAS achievement with 24% and 31% increments, respectively, and anti-SSA single-positive SjD showed no definite effect. The most affected target component was normal serology, with hazard ratios of 0.64–0.85 for overlapping SjD and anti-SSA/SSB double-positive SjD, and 1.15–1.24 for SjD with baseline hypergammaglobulinemia.

**Conclusions:**

Overlapping SjD facilitated reaching LLDAS yet hampered further RONT, anti-SSA/SSB double-positive SjD acted as a hazardous factor, SjD with baseline hypergammaglobulinemia acted as a protective factor, and anti-SSA single-positive SjD acted as an irrelevant factor for RONT achievement.

## Background

Accumulating evidence has confirmed that treat-to-target strategies greatly reduce organ damage accumulation and subsequent flares in patients with systemic lupus erythematosus (SLE). Treatment aiming to reach remission or lupus low disease activity state (LLDAS), in SLE patients, has been recommended (1). Because of the inherent heterogeneity in patient characteristics and disease manifestations, as well as various influencing factors, the proportion of SLE patients who can achieve remission or LLDAS varies greatly among reports. Thus, clarifying the effects of these factors on treatment outcomes is expected to optimize therapeutic regimens (2, 3).

Sjögren’s disease (SjD) has long been recognized as the most common connective tissue disease co-occurring with SLE, occurring in approximately 6.5% to 14.5% of SLE patients (4–6). Cross-sectional studies have shown that patients with SLE overlapping SjD, who are considered a unique subgroup, exhibit distinct clinical and immunological features (4, 6, 7). Overlapping SjD is usually associated with specific immunological characteristics in SLE patients, such as a high prevalence of anti-SSA and anti-SSB antibodies and hypergammaglobulinemia. However, whether overlapping SjD *per se* and its different subgroups act as protective or detrimental factors for SLE patients in achieving remission or LLDAS remains unclear. Many factors influencing outcomes, including gender, age, SLE duration, baseline disease activity status, therapeutic intensity, and organ involvement, may also inevitably act as confounding factors (8–10).

Therefore, we aimed to explore the impact of overlapping SjD and its immunological characteristics on treatment target achievement in patients with SLE. We used propensity score matching (PSM) to adjust for known confounding factors.

## Methods

### Study subjects

The current study was conducted based on the Peking University First Hospital SLE (PKUFHS) cohort, which has been established since 2007. Patients who fulfilled the 1997 American College of Rheumatology (ACR), or 2012 Systemic Lupus International Collaborating Clinics (SLICC), classification criteria for SLE, were aged ≥18 years, and had at least three clinic visits were enrolled (11, 12). SjD was diagnosed using the 2016 ACR/European Alliance of Associations for Rheumatology (EULAR) classification criteria (13). Their medical records through June 2019 were reviewed.

Gender, age of onset, disease duration, baseline therapeutic regimen details, SLE Disease Activity Index (SLEDAI), Physician Global Assessment (PGA), organ involvement (e.g., renal, hematological, neuropsychiatric, and hepatic), serum immunoglobulin G (IgG) level, and the status of clinical or complete remission on treatment (RONT) or LLDAS at each visit were extracted (14, 15). LLDAS, RONT, and relevant components were set as outcomes. The study was approved by the institutional research ethics committee, and all patients provided informed consent for their medical records to be collected.

### Definitions of SjD immunological characteristics

Both anti-SSA and anti-SSB antibodies were detected using an immunoblotting assay. Hypergammaglobulinemia was defined as serum IgG > 16.85 g/L, measured by rate nephelometry. In patients with SLE overlapping SjD, the immunological features of SjD were categorized into three subgroups, namely, anti-SSA single-positive SjD, anti-SSA/SSB double-positive SjD, and SjD with baseline hypergammaglobulinemia. SLE patients without SjD were called the No-SjD group.

### RONT and LLDAS definitions

Definition of Remission In SLE (DORIS) was used to define remission, including clinical SLEDAI = 0 with PGA < 0.5, of which RONT allowed prednisone 5 mg/d at most and maintenance immunosuppressants. Complete RONT further requires normal serology (anti-dsDNA and complements) (8). LLDAS was defined as SLEDAI ≤ 4, with no activity score in cardiopulmonary, renal, central nervous system, vasculitis, or gastrointestinal involvement or hemolytic anemia; no new features compared with the last visit; PGA ≤ 1; prednisone dose ≤ 7.5 mg/d; and well-tolerated maintenance doses of immunosuppressants (14). The status of clinical/complete RONT, LLDAS, and their components was evaluated at each visit. To describe the overall condition, the following were calculated: the proportion of patients reaching the target range at least once, the accumulated time spent within the target range, and the time to first achievement of the target. These metrics were stratified by SjD immunological characteristics of interest.

### The PSM method

To control for confounders affecting RONT or LLDAS achievement in SLE patients, both known and potential variables, including gender, age of onset, SLE duration, baseline disease activity, baseline therapeutic intensity, renal involvement, hematological involvement, neuropsychiatric involvement, and hepatic involvement, were included in the logistic regression model to estimate propensity scores. Standardized mean differences were used to confirm that the groups were adequately matched. Specifically, baseline therapeutic intensity was adopted and demonstrated to be effective in the Hopkins cohort in simplifying heterogeneous therapeutic regimens. Low therapeutic intensity was defined as daily prednisone ≤ 5 mg or equivalent without immunosuppressants, while baseline disease activity was defined according to LLDAS (8). For each SLE patient with a specific immunological feature, propensity score-matched patients in the No-SjD group were selected using a 1:3 nearest neighbor matching algorithm.

### Statistical analysis

A Cox proportional hazards model with recurrent event data was used to analyze the effects of SjD and its immunological characteristics on the attainment of clinical and complete RONT, as well as LLDAS, which could be achieved multiple times during follow-up. Time-varying treatment variables after baseline were also included in the Cox model. The analysis was performed in both unmatched and matched cohorts to estimate the unadjusted and adjusted effects of SjD and its immunological characteristics, respectively. Additionally, subgroup analyses stratified by the aforementioned confounders were conducted.

All outcomes were presented as hazard ratios (HRs) with 95% confidence intervals (CIs). Values were presented as n (%) for categorical variables and median [interquartile range (IQR)] for numerical variables. Two-sided p-values were used, with values of 0.05 or less considered statistically significant. All statistical analyses were conducted using Stata (version 14.0).

## Results

### General features of SLE with/without SjD and its immunological characteristics

A total of 626 SLE patients with 9,415 visits were enrolled in the study. The median follow-up period was 52 months. Among the 626 patients, 77 (12.3%) were recognized as having SLE with overlapping SjD, 35 (5.6%) as having anti-SSA single-positive SjD, 34 (5.4%) as having anti-SSA/anti-SSB double-positive SjD, and 51 (8.1%) as having SjD with baseline hypergammaglobulinemia. Among the 69 total anti-SSA-positive SjD patients, 35 (50.7%) showed single anti-SSA positivity, and the rest showed double positivity for anti-SSA and anti-SSB. Except for the SLE patients with double-positive SjD, the median age at onset of those with overlapping SjD and all other immunological subgroups was at least 10 years older, with a significantly higher female predominance than the No-SjD group. In addition, a significantly higher proportion of hematological involvement was observed in SLE patients with overlapping SjD and those with anti-SSA single-positive SjD (**Supplementary Table S1**).

### Impact of SjD and its immunological characteristics on RONT/LLDAS

In general, the occurrence of LLDAS, clinical RONT, and complete RONT at least once in SLE patients with baseline hypergammaglobulinemia was significantly more frequent than in the No-SjD group (80.4% vs. 64.7%, 56.9% vs. 39.7%, and 52.9% vs. 34.8%, respectively). The proportion of time maintained in LLDAS and in clinical and complete RONT among SjD patients with baseline hypergammaglobulinemia, as well as in complete RONT among anti-SSA single-positive SjD patients, showed a similar tendency. Regarding SLE patients with overlapping SjD (including those with anti-SSA single-positive and anti-SSA/SSB double-positive SjD), the time to first RONT was slightly longer than that in the No-SjD group (**Table 1**).

**Table 1:** Low disease activity/remission achievement in SLE patients with/without SjD features.

	SLE without SjD (n = 549)	SLE with SjD features			
		SjD (n = 77)	Anti-SSA single-positive SjD (n = 35)	Anti-SSA/B-double-positive SjD (n = 34)	SjD with HG (n = 51)
LLDAS at least once, n (%)	355 (64.7)	58 (75.3)	27 (77.1)	25 (73.5)	41 (80.4)^*^
Time to first LLDAS, median (IQR) months	20.0 (12.0, 33.0)	19.5 (15.0, 30.0)	20.0 (10.0, 34.0)	18.0 (16.0, 31.0)	18.0 (14.0, 26.0)
Time ratio on LLDAS during follow-up, median (IQR) %	18.2 (0.0, 52.1)	25.0 (0.0, 53.3)	25.5 (0.0, 55.7)	23.2 (0.0, 61.8)	34.6 (12.5, 60.0)^*^
Clinical RONT at least once, n (%)	218 (39.7)	37 (48.1)	19 (54.3)	14 (41.2)	29 (56.9)^*^
Time to first clinical RONT, median (IQR) months	21.0 (14.0, 38.0)	23.0 (16.0, 35.0)	23.0 (14.0, 34.0)	26.5 (16.0, 37.0)	22.0 (15.0, 31.0)
Time ratio on clinical RONT during follow-up, median (IQR) %	0.0 (0.0, 20.2)	0.0 (0.0, 25.0)	11.2 (0.0, 32.6)	0.0 (0.0, 23.1)	5.6 (0.0, 36.2)^*^
Complete RONT at least once, n (%)	191 (34.8)	35 (45.5)	19 (54.3)^*^	12 (35.3)	27 (52.9)^*^
Time to first complete RONT, median (IQR) months	22.0 (14.0, 38.0)	24.0 (15.0, 37.0)	23.0 (14.0, 35.0)	30.0 (18.0, 38.0)	23.0 (14.0, 35.0)
Time ratio on complete RONT during follow-up, median (IQR) %	0.0 (0.0, 12.5)	0.0 (0.0, 21.7)	8.3 (0.0, 25.0)^*^	0.0 (0.0, 18.2)	0.0 (0.0, 32.6)^*^

Values are presented as median (IQR) for numerical variables or n (%) for categorical variables. *Statistically significant at the level of 0.05.

SLE, systemic lupus erythematosus; SjD, Sjögren's disease; HG, hypergammaglobulinemia; LLDAS, lupus low disease activity state; RONT, remission on treatment; IQR, interquartile range.

The groups of interest were well balanced with the No-SjD group after PSM based on the aforementioned known confounders (**Supplementary Table S1**). Independent of known confounders, overlapping SjD was associated with a 24% higher probability of reaching LLDAS, a 29% lower probability of reaching clinical RONT, and a 38% lower probability of reaching complete RONT. Anti-SSA/SSB double-positive SjD reduced the probability of reaching RONT and achieving normal serology to approximately 50% and 65%, respectively, compared with those of the No-SjD group. In contrast, SjD with hypergammaglobulinemia was associated with a 12% to 31% higher probability of achieving LLDAS, complete RONT, and its component achievements; anti-SSA single-positive SjD showed no significant impact (**Table 2**).

**Table 2:** Hazard ratios for probability of reaching low disease activity or remission associated with overlapping SjD or its features in SLE patients.

	Overlapping SjD		Anti-SSA single-positive SjD		Anti-SSA/SSB double-positive SjD		Overlapping SjD with baseline HG	
	Unmatched	Matched	Unmatched	Matched	Unmatched	Matched	Unmatched	Matched
LLDAS	1.11 (0.98–1.24)	1.24 (1.08–1.44)^†^	1.12 (0.96–1.30)	1.17 (0.94–1.44)	0.99 (0.83–1.20)	0.95 (0.78–1.16)	1.34 (1.16–1.54)^†^	1.31 (1.12–1.54)^†^
SLEDAI ≤ 4	1.11 (1.03–1.19)^†^	1.15 (1.05–1.26)^†^	1.02 (0.92–1.13)	0.95 (0.83–1.08)	1.11 (0.99–1.24)	1.05 (0.93–1.19)	1.22 (1.11–1.33)^†^	1.12 (1.01–1.24)^*^
No new feature	1.01 (0.93–1.08)	1.03 (0.94–1.13)	0.91 (0.82–1.01)	0.95 (0.83–1.09)	1.03 (0.92–1.15)	0.96 (0.85–1.08)	1.09 (1.00–1.20)	1.07 (0.96–1.18)
No active organ involvement	1.08 (1.00–1.17)^*^	1.08 (0.98–1.18)	1.03 (0.93–1.13)	1.00 (0.88–1.14)	1.05 (0.93–1.18)	1.00 (0.88–1.13)	1.17 (1.06–1.28)^†^	1.05 (0.94–1.16)
Clinical RONT	1.03 (0.86–1.23)	0.71 (0.55–0.92)^†^	1.29 (1.04–1.58)^*^	0.75 (0.53–1.06)	0.63 (0.45–0.88)^†^	0.50 (0.35–0.73)^†^	1.39 (1.14–1.71)^†^	1.25 (0.98–1.59)
Complete RONT	1.05 (0.87–1.27)	0.62 (0.46–0.83)^†^	1.34 (1.08–1.67)^†^	0.84 (0.58–1.22)	0.59 (0.41–0.85)^†^	0.47 (0.31–0.70)^†^	1.49 (1.21–1.84)^†^	1.30 (1.01–1.68)^*^
cSLEDAI = 0	1.16 (1.07–1.26)^†^	1.17 (1.06–1.30)^†^	1.05 (0.94–1.17)	0.98 (0.85–1.14)	1.15 (1.02–1.30)^*^	1.12 (0.98–1.28)	1.26 (1.14–1.40)^†^	1.16 (1.04–1.29)^†^
Normal serology	1.12 (1.01–1.24)^*^	0.85 (0.73–0.98)^*^	1.30 (1.14–1.47)^†^	0.99 (0.82–1.20)	0.85 (0.71–1.01)	0.64 (0.52–0.78)^†^	1.27 (1.12–1.44)^†^	1.24 (1.07–1.43)^†^
Negative anti-dsDNA	1.07 (0.97–1.17)	0.84 (0.74–0.96)^†^	1.25 (1.11–1.40)^†^	0.96 (0.82–1.13)	0.82 (0.70–0.97)^*^	0.65 (0.54–0.78)^†^	1.21 (1.08–1.35)^†^	1.15 (1.01–1.31)^*^
PGA ≤ 1	1.15 (1.07–1.24)^†^	1.12 (1.01–1.24)^*^	1.06 (0.95–1.17)	1.02 (0.88–1.18)	1.13 (1.01–1.27)^*^	1.05 (0.92–1.20)	1.27 (1.16–1.40)^†^	1.23 (1.10–1.37)^†^
Pred ≤ 5 mg/d	1.02 (0.95–1.10)	1.06 (0.97–1.15)	0.94 (0.86–1.04)	0.96 (0.84–1.08)	1.04 (0.93–1.15)	0.98 (0.87–1.10)	1.10 (1.01–1.20)^*^	1.06 (0.96–1.17)
No immunosuppressant	1.03 (0.92–1.16)	1.19 (1.04–1.37)^*^	1.10 (0.95–1.27)	1.16 (0.94–1.43)	0.88 (0.73–1.05)	0.83 (0.68–1.01)	1.24 (1.09–1.42)^†^	1.17 (1.00–1.36)^*^

Values are presented as hazard ratio (95% CI) for reaching LLDAS and clinical/complete RONT and relevant components associated with SjD features of interest in SLE patients. Unmatched cohort refers to whole sample (n = 626), and matched cohort refers to propensity score-matched patients divided by different SjD features after correcting for gender, age of onset, SLE duration, baseline therapeutic intensity, baseline disease activity, and renal, hematological, neuropsychiatric, and hepatic involvement.

SLE, systemic lupus erythematosus; SjD, Sjögren's disease; HG, hypergammaglobulinemia; LLDAS, lupus low disease activity state; RONT, remission on treatment; IQR, interquartile range; cSLEDAI, clinical Systemic Lupus Erythematosus Disease Activity Index; PGA, Physician Global Assessment; Pred, prednisone dose or equivalent.

^*^Statistically significant at the level of 0.05.

^†^Statistically significant at the level of 0.01.

Regarding the components, normal serology was affected in patients with overlapping SjD, double-positive SjD, and SjD with baseline hypergammaglobulinemia. Both achieving a PGA ≤ 1 and the absence of immunosuppressant treatment were 12% to 23% more attainable in SLE patients with overlapping SjD and SjD with baseline hypergammaglobulinemia.

### Sensitivity analyses

The impact of specific immunological characteristics of SjD on the achievement of clinical or complete RONT and LLDAS was mostly consistent across subgroups of SLE patients stratified by critical confounders. Similarly, the target components in SLE patients showed consistent results in these subgroups (**Table 3**).

**Table 3:** Hazard ratios for probability of reaching remission or low disease activity associated with overlapping SjD or its features stratified by critical confounding factors in SLE patients.

	Gender	Age of onset	Duration	Low treatment	Low activity	Renal involvement	Hematological involvement	NP-SLE
LLDAS								
Overlapping SjD	1.25 (1.08–1.44)^†^	1.10 (0.95–1.28)	1.20 (1.04–1.39)^*^	1.25 (1.08–1.44)^†^	1.22 (1.05–1.41)^†^	1.22 (1.05–1.42)^†^	1.41 (1.22–1.63)^†^	1.17 (1.01–1.36)^*^
Anti-SSA single-positive SjD	1.16 (0.94–1.44)	1.00 (0.79–1.26)	1.16 (0.93–1.45)	1.14 (0.92–1.41)	1.19 (0.96–1.48)	1.14 (0.92–1.43)	1.37 (1.12–1.68)^†^	1.19 (0.97–1.47)
Anti-SSA/SSB double-positive SjD	0.93 (0.76–1.14)	0.91 (0.74–1.13)	0.98 (0.80–1.20)	0.95 (0.78–1.16)	0.87 (0.71–1.07)	0.94 (0.76–1.15)	1.07 (0.88–1.31)	0.93 (0.76–1.14)
Overlapping SjD with baseline HG	1.31 (1.12–1.54)^†^	1.24 (1.05–1.46)^*^	1.32 (1.13–1.55)^†^	1.30 (1.11–1.53)^†^	1.29 (1.10–1.51)^†^	1.32 (1.13–1.55)^†^	1.52 (1.29–1.80)^†^	1.28 (1.09–1.50)^†^
SLEDAI ≤ 4								
Overlapping SjD	1.16 (1.05–1.27)^†^	1.09 (1.00–1.20)	1.13 (1.04–1.24)^†^	1.15 (1.05–1.26)^†^	1.13 (1.04–1.24)^†^	1.09 (0.99–1.19)	1.21 (1.11–1.32)^†^	1.13 (1.03–1.24)^*^
Anti-SSA single-positive SjD	0.95 (0.83–1.08)	0.90 (0.78–1.03)	0.93 (0.81–1.07)	0.94 (0.82–1.07)	0.96 (0.84–1.09)	0.96 (0.84–1.10)	0.98 (0.86–1.12)	0.95 (0.84–1.08)
Anti-SSA/SSB double-positive SjD	1.05 (0.93–1.18)	1.05 (0.93–1.19)	1.07 (0.95–1.20)	1.05 (0.93–1.19)	1.03 (0.91–1.16)	1.04 (0.92–1.18)	1.10 (0.98–1.24)	1.04 (0.92–1.18)
Overlapping SjD with baseline HG	1.12 (1.01–1.23)^*^	1.08 (0.98–1.19)	1.12 (1.02–1.24)^*^	1.12 (1.01–1.23)^*^	1.12 (1.01–1.24)^*^	1.12 (1.02–1.24)^*^	1.20 (1.09–1.33)^†^	1.10 (0.99–1.22)
No new SLE feature								
Overlapping SjD	1.03 (0.94–1.13)	0.99 (0.91–1.09)	1.03 (0.95–1.13)	1.03 (0.94–1.13)	1.02 (0.93–1.12)	1.00 (0.92–1.10)	1.07 (0.98–1.17)	1.02 (0.93–1.11)
Anti-SSA single-positive SjD	0.94 (0.82–1.08)	0.90 (0.79–1.04)	0.94 (0.82–1.08)	0.94 (0.82–1.07)	0.95 (0.83–1.09)	1.00 (0.88–1.15)	0.99 (0.86–1.13)	0.96 (0.84–1.09)
Anti-SSA/SSB double-positive SjD	0.96 (0.85–1.08)	0.96 (0.85–1.09)	0.97 (0.86–1.09)	0.96 (0.85–1.08)	0.94 (0.84–1.07)	0.96 (0.85–1.09)	0.99 (0.88–1.11)	0.96 (0.85–1.08)
Overlapping SjD with baseline HG	1.06 (0.96–1.18)	1.04 (0.94–1.15)	1.08 (0.98–1.20)	1.07 (0.96–1.18)	1.08 (0.97–1.19)	1.07 (0.97–1.19)	1.14 (1.03–1.27)^*^	1.07 (0.96–1.18)
No active organ involvement								
Overlapping SjD	1.08 (0.98–1.18)	1.05 (0.95–1.15)	1.06 (0.97–1.16)	1.08 (0.98–1.18)	1.07 (0.97–1.17)	1.00 (0.91–1.09)	1.12 (1.02–1.22)^*^	1.06 (0.96–1.16)
Anti-SSA single-positive SjD	1.00 (0.88–1.14)	0.95 (0.83–1.08)	0.98 (0.86–1.12)	0.99 (0.87–1.12)	1.01 (0.89–1.14)	0.99 (0.87–1.12)	1.03 (0.91–1.17)	1.00 (0.88–1.13)
Anti-SSA/SSB double-positive SjD	0.99 (0.88–1.12)	1.00 (0.88–1.13)	1.00 (0.88–1.12)	1.00 (0.88–1.13)	0.97 (0.86–1.10)	0.98 (0.87–1.11)	1.02 (0.91–1.16)	0.98 (0.87–1.11)
Overlapping SjD with baseline HG	1.04 (0.94–1.16)	1.02 (0.92–1.13)	1.05 (0.95–1.16)	1.04 (0.94–1.16)	1.05 (0.95–1.17)	1.01 (0.91–1.13)	1.10 (0.99–1.22)	1.03 (0.92–1.14)
Clinical RONT								
Overlapping SjD	0.70 (0.54–0.91)^†^	0.66 (0.51–0.85)^†^	0.66 (0.51–0.86)^†^	0.72 (0.56–0.93)^*^	0.68 (0.53–0.88)^†^	0.75 (0.58–0.97)^*^	0.82 (0.63–1.06)	0.66 (0.51–0.85)^†^
Anti-SSA single-positive SjD	0.74 (0.52–1.05)	0.81 (0.55–1.18)	0.76 (0.54–1.07)	0.75 (0.53–1.06)	0.75 (0.53–1.07)	0.74 (0.51–1.06)	0.85 (0.60–1.21)	0.79 (0.56–1.12)
Anti-SSA/SSB double-positive SjD	0.49 (0.34–0.71)^†^	0.51 (0.35–0.75)^†^	0.58 (0.40–0.84)^†^	0.50 (0.35–0.73)^†^	0.43 (0.30–0.62)^†^	0.49 (0.34–0.72)^†^	0.58 (0.39–0.85)^†^	0.49 (0.33–0.71)^†^
Overlapping SjD with baseline HG	1.25 (0.98–1.58)	1.16 (0.92–1.48)	1.21 (0.95–1.55)	1.25 (0.98–1.59)	1.24 (0.98–1.57)	1.32 (1.04–1.67)^*^	1.36 (1.06–1.75)^*^	1.21 (0.95–1.54)
Complete RONT								
Overlapping SjD	0.61 (0.46–0.82)^†^	0.58 (0.43–0.77)^†^	0.55 (0.41–0.75)^†^	0.63 (0.47–0.84)^†^	0.59 (0.44–0.79)^†^	0.68 (0.51–0.91)^†^	0.70 (0.52–0.94)^*^	0.57 (0.43–0.77)^†^
Anti-SSA single-positive SjD	0.84 (0.58–1.22)	0.79 (0.52–1.20)	0.84 (0.58–1.22)	0.85 (0.58–1.22)	0.86 (0.59–1.24)	0.91 (0.62–1.35)	0.90 (0.62–1.31)	0.88 (0.61–1.28)
Anti-SSA/SSB double-positive SjD	0.46 (0.30–0.69)^†^	0.46 (0.30–0.70)^†^	0.48 (0.31–0.74)^†^	0.47 (0.31–0.70)^†^	0.39 (0.26–0.59)^†^	0.44 (0.29–0.68)^†^	0.50 (0.33–0.77)^†^	0.46 (0.31–0.70)^†^
Overlapping SjD with baseline HG	1.30 (1.01–1.68)^*^	1.21 (0.94–1.56)	1.25 (0.96–1.62)	1.31 (1.01–1.69)^*^	1.30 (1.01–1.68)^*^	1.41 (1.09–1.81)^†^	1.36 (1.04–1.78)^*^	1.28 (0.99–1.65)
Normal serology								
Overlapping SjD	0.84 (0.72–0.98)^*^	0.83 (0.71–0.96)^*^	0.81 (0.70–0.94)^†^	0.86 (0.74–0.99)^*^	0.83 (0.72–0.97)^*^	0.92 (0.79–1.07)	0.89 (0.77–1.04)	0.84 (0.72–0.97)^*^
Anti-SSA single-positive SjD	0.99 (0.82–1.20)	0.93 (0.76–1.13)	0.98 (0.81–1.18)	1.00 (0.83–1.20)	1.02 (0.85–1.23)	1.08 (0.90–1.31)	0.98 (0.81–1.19)	0.99 (0.82–1.20)
Anti-SSA/SSB double-positive SjD	0.63 (0.51–0.78)^†^	0.64 (0.52–0.79)^†^	0.64 (0.52–0.79)^†^	0.64 (0.52–0.78)^†^	0.60 (0.49–0.74)^†^	0.64 (0.52–0.78)^†^	0.67 (0.54–0.82)^†^	0.63 (0.51–0.78)^†^
Overlapping SjD with baseline HG	1.24 (1.07–1.43)^†^	1.20 (1.03–1.38)^*^	1.21 (1.05–1.40)^†^	1.24 (1.07–1.43)^†^	1.26 (1.09–1.45)^†^	1.31 (1.14–1.52)^†^	1.29 (1.11–1.50)^†^	1.24 (1.07–1.44)^†^
Negative anti-dsDNA								
Overlapping SjD	0.83 (0.73–0.95)^†^	0.83 (0.73–0.94)^†^	0.84 (0.74–0.95)^†^	0.85 (0.75–0.96)^*^	0.83 (0.73–0.95)^†^	0.87 (0.77–0.99)^*^	0.89 (0.78–1.01)	0.83 (0.73–0.95)^†^
Anti-SSA single-positive SjD	0.96 (0.81–1.12)	0.96 (0.81–1.14)	0.96 (0.82–1.13)	0.96 (0.82–1.13)	0.97 (0.83–1.14)	1.00 (0.85–1.17)	0.97 (0.82–1.14)	0.95 (0.80–1.11)
Anti-SSA/SSB double-positive SjD	0.65 (0.54–0.77)^†^	0.67 (0.56–0.80)^†^	0.69 (0.58–0.82)^†^	0.65 (0.54–0.78)^†^	0.62 (0.52–0.75)^†^	0.66 (0.55–0.79)^†^	0.68 (0.57–0.82)^†^	0.65 (0.54–0.78)^†^
Overlapping SjD with baseline HG	1.15 (1.01–1.31)^*^	1.13 (0.99–1.29)	1.15 (1.01–1.31)^*^	1.16 (1.01–1.32)^*^	1.17 (1.03–1.33)^*^	1.21 (1.07–1.38)^†^	1.20 (1.05–1.37)^†^	1.16 (1.02–1.33)^*^
Normal complement								
Overlapping SjD	1.13 (1.02–1.25)^*^	1.05 (0.95–1.17)	1.08 (0.98–1.20)	1.13 (1.02–1.24)^*^	1.11 (1.01–1.23)^*^	1.14 (1.03–1.26)^*^	1.16 (1.05–1.28)^†^	1.11 (1.00–1.23)^*^
Anti-SSA single-positive SjD	1.01 (0.87–1.17)	0.87 (0.75–1.02)	0.99 (0.86–1.15)	1.02 (0.89–1.18)	1.04 (0.90–1.20)	1.10 (0.95–1.27)	1.04 (0.90–1.20)	1.03 (0.89–1.19)
Anti-SSA/SSB double-positive SjD	1.04 (0.92–1.19)	1.03 (0.90–1.18)	1.03 (0.90–1.17)	1.05 (0.92–1.20)	1.02 (0.90–1.17)	1.05 (0.92–1.20)	1.07 (0.94–1.23)	1.04 (0.91–1.18)
Overlapping SjD with baseline HG	1.22 (1.09–1.36)^†^	1.17 (1.05–1.31)^†^	1.21 (1.08–1.34)^†^	1.23 (1.10–1.37)^†^	1.24 (1.11–1.38)^†^	1.25 (1.12–1.39)^†^	1.25 (1.12–1.40)^†^	1.23 (1.10–1.37)^†^
PGA ≤ 1								
Overlapping SjD	1.06 (0.98–1.16)	1.02 (0.94–1.11)	1.05 (0.96–1.14)	1.06 (0.97–1.15)	1.05 (0.96–1.14)	1.03 (0.94–1.12)	1.10 (1.01–1.20)^*^	1.03 (0.94–1.12)
Anti-SSA single-positive SjD	0.95 (0.84–1.08)	0.90 (0.79–1.02)	0.94 (0.83–1.07)	0.94 (0.83–1.07)	0.96 (0.85–1.09)	1.00 (0.88–1.13)	0.98 (0.87–1.11)	0.96 (0.85–1.08)
Anti-SSA/SSB double-positive SjD	0.98 (0.87–1.10)	0.98 (0.87–1.10)	0.97 (0.87–1.09)	0.98 (0.87–1.10)	0.96 (0.86–1.08)	0.98 (0.88–1.10)	1.01 (0.90–1.13)	0.97 (0.87–1.09)
Overlapping SjD with baseline HG	1.05 (0.96–1.16)	1.03 (0.94–1.14)	1.07 (0.97–1.17)	1.06 (0.96–1.17)	1.07 (0.97–1.18)	1.06 (0.96–1.17)	1.13 (1.02–1.24)^*^	1.04 (0.94–1.15)
Pred ≤ 5 mg/d								
Overlapping SjD	1.20 (1.04–1.38)^*^	1.05 (0.91–1.21)	1.16 (1.00–1.33)^*^	1.20 (1.04–1.38)^*^	1.17 (1.01–1.34)^*^	1.18 (1.03–1.36)^*^	1.31 (1.14–1.51)^†^	1.12 (0.98–1.29)
Anti-SSA single-positive SjD	1.15 (0.93–1.42)	1.03 (0.83–1.28)	1.16 (0.94–1.44)	1.12 (0.91–1.39)	1.19 (0.97–1.47)	1.14 (0.92–1.42)	1.33 (1.09–1.63)^†^	1.20 (0.98–1.47)
Anti-SSA/SSB double-positive SjD	0.81 (0.67–0.99)^*^	0.78 (0.64–0.97)^*^	0.84 (0.69–1.02)	0.83 (0.68–1.01)	0.76 (0.63–0.93)^†^	0.83 (0.68–1.01)	0.89 (0.74–1.09)	0.81 (0.67–0.98)^*^
Overlapping SjD with baseline HG	1.16 (1.00–1.36)	1.10 (0.94–1.29)	1.18 (1.01–1.37)^*^	1.16 (1.00–1.35)	1.15 (0.99–1.33)	1.20 (1.03–1.39)^*^	1.30 (1.11–1.52)^†^	1.13 (0.97–1.32)
No immunosuppressant								
Overlapping SjD	1.02 (0.88–1.17)	0.93 (0.80–1.08)	0.98 (0.84–1.13)	1.05 (0.91–1.21)	1.01 (0.88–1.17)	0.94 (0.81–1.09)	1.10 (0.95–1.27)	0.99 (0.86–1.15)
Anti-SSA single-positive SjD	1.05 (0.88–1.25)	0.94 (0.78–1.13)	1.04 (0.87–1.24)	1.03 (0.87–1.23)	1.08 (0.91–1.29)	0.89 (0.74–1.08)	1.11 (0.92–1.33)	1.09 (0.91–1.29)
Anti-SSA/SSB double-positive SjD	0.63 (0.51–0.78)^†^	0.59 (0.47–0.74)^†^	0.64 (0.52–0.79)^†^	0.64 (0.52–0.79)^†^	0.61 (0.50–0.76)^†^	0.61 (0.50–0.76)^†^	0.69 (0.56–0.85)^†^	0.63 (0.51–0.77)^†^
Overlapping SjD with baseline HG	1.07 (0.93–1.24)	0.99 (0.86–1.15)	1.08 (0.93–1.24)	1.08 (0.93–1.24)	1.08 (0.93–1.25)	1.03 (0.89–1.19)	1.23 (1.05–1.42)^†^	1.04 (0.90–1.20)

Values are presented as stratified hazard ratio (95% CI) associated with SjD features for reaching LLDAS, clinical/complete RONT, and relative components in SLE patients according to gender, age of onset, duration, baseline therapeutic intensity, disease activity, lupus nephritis, hematological involvement, and NP-SLE.

SLE, systemic lupus erythematosus; NP-SLE, neuropsychiatric systemic lupus erythematosus; SjD, Sjögren's disease; HG, hypergammaglobulinemia; LLDAS, lupus low disease activity state; RONT, remission on treatment; IQR, interquartile range.

^*^Statistically significant at the level of 0.05.

^†^Statistically significant at the level of 0.01.

## Discussion

Our study examined the influence of SjD immunological characteristics on the achievement of remission and LLDAS, as well as on related clinical components in SLE patients. This analysis was conducted using the PSM method in a real-world cohort with a median follow-up of 4.3 years.

Overall, anti-SSA-positive SjD patients were either positive for anti-SSA alone or positive for both anti-SSA and anti-SSB. This confirms that isolated positivity for anti-SSB alone is rare in SjD patients (16). The comparison of general characteristics related to critical influencing factors before matching indicated that SLE patients with different SjD immunological characteristics tended to be older at the onset of SLE, showed a significant predominance of female patients, and had more frequent hematological involvement. These findings are largely consistent with those of De Marchi G et al. and Jiménez PP et al. (17, 18). All these characteristics may collectively contribute to a variable probability of reaching remission, making the exploration of their net effect important but challenging.

Overlapping SjD and its immunological characteristics showed differing effects on the achievement of clinical and complete RONT and LLDAS in SLE patients. The hindrance to achieving further remission beyond LLDAS and the inconsistent results observed in composite clinical outcomes and their relevant components in cases of overlapping SjD suggest that opposing mechanisms may be involved. These results suggest that SLE patients with SjD who have reached LLDAS should continue to be closely monitored. Intensive treatment should be given to achieve clinical remission to reduce the risk of irreversible organ damage and steroid-related adverse events. Correspondingly, both the adverse impact of SjD patients double-positive for anti-SSA and anti-SSB antibodies and the protective effect of SjD patients presenting with baseline hypergammaglobulinemia were confirmed.

Analyzing the impact on the components of RONT and LLDAS could further facilitate understanding the correlation between SjD characteristics and SLE. On the one hand, SjD with baseline hypergammaglobulinemia facilitated achieving targets for all the components of RONT, indicating a more controllable course. On the other hand, despite the insignificant influence of single anti-SSA positivity, double positivity for anti-SSA and anti-SSB antibodies impeded achieving normal serological markers. The possible explanations for these contrasting impacts are as follows.

First, the genetic link between the diseases is significant. Increasing research on the genetic connections has provided a clearer understanding of SLE and SjD. Tumor necrosis factor alpha inducible protein 3 (TNFAIP3) encodes the deubiquitinase A20 and acts as a negative regulator of the inflammatory cascade. Moreover, the rs6920220 single-nucleotide polymorphism of TNFAIP3 has been shown to be associated with susceptibility to both SLE and SjD (19). Recently, Kamitaki et al. reported that the decreased copy number of the *C4* gene was associated with a sevenfold higher risk for SLE and a 16-fold higher risk for SjD (20).

Second, there is crosstalk among autoantibodies. On the one hand, SSA has been shown to directly participate in systemic inflammation by promoting immune complex formation and subsequent cytokine production. Ro60 was considered a quality checkpoint of RNAs by tagging misfolded RNAs for further degradation and epitope spreading. Meanwhile, Ro52, as an E3 ubiquitin ligase, negatively regulates the interferon-mediated immune response (21, 22). On the other hand, the nucleic acid components associated with SSB were transcripts synthesized by RNA polymerase III. These transcripts may interact with small RNAs, such as those encoded by Epstein–Barr virus or hepatitis C virus, which can induce autoantibody production (23–25). Under certain circumstances, such as apoptosis, SSA and SSB antigens can be translocated to the cell surface, where they are bound by anti-SSA/SSB antibodies. This binding initiates antibody-dependent cell-mediated cytotoxicity and induces the production of new autoantibodies (26). Furthermore, Ro60 and SSB transiently cooperate, facilitating antigen spreading to other associated autoantigens once the immune response is evoked (27, 28). Such mechanisms laid the foundation for the multiplicative effect of anti-SSB antibody positivity on anti-SSA positivity alone, as observed in our cohorts.

Third, the presence of baseline hypergammaglobulinemia—an elevated level of immunoglobulins in the blood—often indicates more active immune abnormalities in patients with SjD. This activity may increase the likelihood of reversing active lesions. Clinically, doctors prefer to choose more aggressive therapeutic agents for patients with elevated globulin levels, including traditional immunosuppressive drugs and biological agents that are more effective in inducing B-cell depletion and presumably help SLE patients achieve LLDAS more effectively. However, more research is needed to clarify the biological significance of hypergammaglobulinemia in SLE patients, particularly in those with SjD.

The impact of overlapping SjD in SLE patients is not as mild as reported in cross-sectional studies, and it is postulated that these patients are more likely to reach RONT (18). Accordingly, there was discordance between the actual harm caused by SjD immunological features and the PGA. The hazardous net effect verified by the current study can be attributed to the mutual reinforcement of the underlying mechanisms and adequate adjustment for relevant confounding factors. Based on the discussion above, the coexistence of SjD and baseline hypergammaglobulinemia generally facilitates achieving treatment targets, while anti-SSA/SSB double-positive SjD significantly delays SLE patients from achieving treatment targets across nearly all composite outcomes and their relevant components. It would be appropriate for these patients to monitor their disease activity more closely and to intensify treatment to help them achieve clinical remission.

The limitations of the study mainly include the following. There was a relatively low frequency of overlapping SjD in our SLE cohort. The study was retrospective and conducted in a single-center cohort. There was potential for referral bias at a tertiary center and its impact on the observed frequency of overlap SjD. However, the data were complete and representative, and several articles based on this cohort have been published (29, 30).

## Conclusions

Overlapping SjD independently prevented SLE patients from achieving further RONT from LLDAS. Among these factors, anti-SSA/SSB double positivity exhibited a hazardous effect, whereas baseline hypergammaglobulinemia acted as a protective factor.

## Data Availability

The original contributions presented in the study are included in the article/**Supplementary Material**. Further inquiries can be directed to the corresponding author.
