# Supplementary material for: Impact of Sjögren’s disease and its immunological characteristics on reaching remission or low disease activity state in systemic lupus erythematosus patients: a propensity score-matched longitudinal study
Source: Front Immunol. 2025 Sep 29;16:1639252. doi: 10.3389/fimmu.2025.1639252 (PMC12515826; doi:10.3389/fimmu.2025.1639252)
Supplement: Supplementary file 1 [file Supplementaryfile1.docx]

| Table S Critical Characteristics in the Unmatched and Propensity-Score Matched Cohorts | | | | | | | | |
| --- | --- | --- | --- | --- | --- | --- | --- | --- |
|  | Female  n (%) | Age of onset  median (IQR) years | SLE duration  median (IQR) months | Low treatment  n (%) | Baseline LLDAS  n (%) | Lupus nephritis  n (%) | Hematological involvement  n (%) | NP-SLE  n (%) |
| Unmatched Cohort |  |  |  |  |  |  |  |  |
| SLE-SjD (n=77) | 75 (97.4) | 39.3 (29.2, 48.3) | 2.0 (0.3, 5.2) | 4 (5.2) | 4 (5.2) | 28 (36.4) | 46 (59.7) | 8 (10.4) |
| SLE without SjD (n=549) | 486 (88.5) | 27.6 (21.1, 36.5) | 2.0 (0.5, 7.1) | 14 (2.6) | 33 (6.0) | 245 (44.6) | 258 (47.0) | 50 (9.1) |
| P | 0.017 | <0.001 | 0.731 | 0.194 | 0.777 | 0.171 | 0.036 | 0.717 |
| Anti-SSA-single-positive SjD (n=35) | 34 (97.1) | 43.9 (30.4, 53.9) | 3.0 (0.8, 8.1) | 4 (11.4) | 3 (8.6) | 13 (37.1) | 23 (65.7) | 3 (8.6) |
| SLE without SjD (n=549) | 486 (88.5) | 27.6 (21.1, 36.5) | 2.0 (0.5, 7.1) | 14 (2.6) | 33 (6.0) | 245 (44.6) | 258 (47.0) | 50 (9.1) |
| P | 0.114 | <0.001 | 0.557 | 0.003 | 0.542 | 0.388 | 0.032 | 0.915 |
| Anti-SSA/SSB-double-positive SjD (n=34) | 33 (97.1) | 31.9 (24.9, 42.1) | 0.5 (0.2, 5.1) | 0 (0.0) | 1 (2.9) | 14 (41.2) | 18 (52.9) | 4 (11.8) |
| SLE without SjD (n=532) | 457 (85.9) | 27.6 (21.1, 36.5) | 2.0 (0.5, 7.1) | 14 (2.6) | 33 (6.0) | 245 (46.1) | 258 (48.5) | 50 (9.4) |
| P | 0.129 | 0.184 | 0.512 | - | 0.733 | 0.595 | 0.543 | 0.621 |
| SLE- SjD with baseline HG (n=51) | 50 (98.0) | 38.7 (29.0, 48.3) | 2.0 (0.2, 5.2) | 3 (5.9) | 3 (5.9) | 17 (33.3) | 30 (58.8) | 5 (9.8) |
| SLE without SjD (n=546) | 486 (89.0) | 27.6 (21.1, 36.5) | 2.0 (0.5, 7.1) | 14 (2.6) | 33 (6.0) | 245 (44.9) | 258 (47.3) | 50 (9.2) |
| P | 0.037 | <0.001 | 0.854 | 0.174 | 0.995 | 0.113 | 0.108 | 0.844 |
| Matched Cohort |  |  |  |  |  |  |  |  |
| SLE- SjD (n=54) | 52 (96.3) | 32.4(24.2, 44.1) | 1.0 (0.2, 5.1) | 1 (1.9) | 2 (3.7) | 23 (42.6) | 32 (59.3) | 5 (9.3) |
| SLE without SjD (n=188) | 179 (95.2) | 28.7 (23.8, 36.2) | 2.0 (0.5, 5.3) | 1 (0.5) | 7 (3.7) | 76 (40.4) | 101 (53.7) | 23 (12.2) |
| P | 0.812 | 0.661 | 0.246 | 0.684 | 0.872 | 0.538 | 0.974 | 0.679 |
| Anti-SSA-single-positive SjD (n=23) | 22 (95.7) | 38.6 (23.9, 48.6) | 3.0 (0.7, 9.8) | 1 (4.3) | 1 (4.3) | 9 (39.1) | 15 (65.2) | 2 (8.7) |
| SLE without SjD (n=86) | 83 (96.5) | 30.4 (25.5, 37.9) | 3.0 (1.0, 7.1) | 0 (0.0) | 4 (4.7) | 34 (39.5) | 49 (57.0) | 13 (15.1) |
| P | 0.798 | 0.894 | 0.371 | 0.323 | 0.752 | 0.698 | 0.920 | 0.206 |
| Anti-SSA/SSB-double-positive SjD (n=29) | 28 (96.6) | 31.2 (24.9, 36.8) | 0.5 (0.2, 5.1) | 0 (0.0) | 1 (3.4) | 12 (52.2) | 18 (62.1) | 2 (6.9) |
| SLE without SjD (n=146) | 145 (99.3) | 29.3 (24.6, 35.5) | 1.7 (0.5, 5.1) | 0 (0.0) | 5 (3.4) | 60 (41.1) | 85 (58.2) | 13 (8.9) |
| P | 1.000 | 0.856 | 0.943 | - | 0.798 | 0.861 | 0.827 | 0.698 |
| SLE- SjD with baseline HG (n=41) | 40 (97.6) | 36.2 (27.2, 46.0) | 1.0 (0.2, 5.1) | 1 (2.4) | 2 (4.9) | 16 (39.0) | 25 (61.0) | 4 (9.8) |
| SLE without SjD (n=173) | 171 (98.8) | 27.3 (21.6, 34.0) | 2.0 (0.5, 7.1) | 1 (0.6) | 5 (2.9) | 67 (38.7) | 93 (53.8) | 25 (14.5) |
| P | 0.320 | 0.443 | 0.195 | 0.566 | 0.861 | 0.358 | 0.941 | 0.610 |

SLE refers to systemic lupus erythematosus; NP-SLE refers to Neuropsychiatric Systemic Lupus Erythematosus; SjD refers to Sjögren's disease; HG refers to hypergammaglobulinemia; LLDAS refers to Lupus Low Disease Activity State; RONT refers to remission on treatment; IQR refers to interquartile ranges.
